# A tribute to Richard L. Maas (1954–2025)

**DOI:** 10.1172/JCI201292

**Published:** 2025-11-17

**Authors:** Alireza Haghighi, Salil A. Lachke, Natasha Y. Frank, Wolfram Goessling, Philip A. Cole

**Affiliations:** 1 Division of Genetics, Department of Medicine, Brigham and Women’s Hospital, Mass General Brigham, and Harvard Medical School, Boston, Massachusetts, USA.; 2 Department of Biological Sciences, University of Delaware, Newark, Delaware, USA.; 3 Department of Internal Medicine, Yale School of Medicine, New Haven, Connecticut, USA.; 4 Department of Biological Chemistry and Molecular Pharmacology, Harvard Medical School, Boston, Massachusetts, USA.

Dr. Richard (Dick) L. Maas, Professor of Medicine at Harvard Medical School, passed away on August 15, 2025, after complications of a longstanding illness, at age 70 (Figure 1). He was a leading physician-scientist and member of the American Society for Clinical Investigation who served as the Division Chief of Genetics at Brigham and Women’s Hospital (BWH) 1999–2021.

Dick was born in Baltimore in 1954, an only child of Louis and Elizabeth Maas, and grew up in Severna Park, Maryland. He graduated from Dartmouth College in 1976 with a degree in chemistry and obtained his MD and PhD at Vanderbilt University in 1984. At Vanderbilt, he carried out his thesis research in the lab of leading clinical pharmacologist Dr. John Oates, studying the biosynthetic pathways of leukotrienes. In this work, Dick synthesized isotopically labeled arachidonic acid analogs as leukotriene precursors. This work led to the discovery of 15-lipoxygenation, defining an alternative pathway to the production of complex leukotrienes (1). This painstaking research involved cutting-edge mass spectrometry and analytical pharmacology that moved the frontier of our understanding of inflammatory mediators. Following his time at Vanderbilt, Dick entered the medicine residency program at BWH in 1984. A fellow resident, Alan Garber, current President of Harvard University, said about Dick that he was “a really wonderful physician with an inquisitive mind who cared deeply about his patients.” Dick’s clinical training was interspersed with postdoctoral work in the Department of Genetics at Harvard Medical School in the lab of Philip Leder, who had recently relocated his lab from the NIH to Harvard. Dick’s research interests pivoted from pharmacology to developmental genetics, and as a postdoc, he helped identify a gene family called the formins, which encodes proteins that are critical for mammalian limb development (2). The formin proteins are now understood to be key regulators of cytoskeleton and actin remodeling.

Dick started his independent research program in 1989 in the newly formed Division of Genetics in the Department of Medicine at BWH. Over the course of 30 years, the Maas laboratory made seminal discoveries in the genetic control of vertebrate organogenesis, having a transformative impact on eye and craniofacial development, while making significant advances on the developmental biology of other organs. Early studies focused on the gene paired box 6 (*PAX6*), which encodes a paired-box homeodomain transcription factor critical for eye development in organisms as diverse as fruit flies and humans. Key studies from the Maas laboratory showed that *PAX6* mutations caused the human ocular birth defect aniridia (partial/complete loss of iris); uncovered its transcriptional activation, alternative splicing, and DNA-binding properties (3); and demonstrated that aspects of its upstream regulation were conserved between insects and mammals. Later studies revealed that Pax6 controls a conserved regulatory network of key transcription factors, belonging to the Eya and Six families, in the eye (4). These findings contributed to a paradigm wherein different combinations of *Pax*, *Eya*, and *Six* genes govern early regulatory events in the development of different organs in vertebrates. Over the years, the Maas laboratory continued to discover new genes critical to eye development. Among others, key upstream regulators of Pax6, namely, the Meis and Pknox1 homeoproteins, were identified (5). In particular, characterization of Pknox1’s role in early lens development demonstrated the importance of differential binding affinities of *cis*-regulatory sites for achieving temporal specificity in enhancer activation, and thereby, for calibrating the dosage of key proteins in development. Further, a surprising discovery that a tudor family protein, Tdrd7 — previously linked primarily to germ cell differentiation — is necessary for lens transparency in human, mouse, and chicken opened a new research area, that of posttranscriptional gene expression control, in relation to cataract (6).

Additionally, the Maas laboratory made significant advances in our understanding of craniofacial development. In 1994, Satokata and Maas showed that mice lacking the homeoprotein msh homeobox 1 (Msx1) failed to form teeth, which were arrested at the early bud stage (7). They also demonstrated that a related transcription factor, Msx2, has a key role in proper formation of bone and of ectodermally derived organs, including the tooth. Dick considered the developing tooth an effective model for understanding epithelial-mesenchymal interactions in organogenesis. His laboratory characterized many pathways in tooth formation and uncovered evidence supportive of a covert population of epithelial stem cells that retain the capacity to induce supernumerary teeth in response to canonical WNT signaling activation.

Beyond his lab’s research, Dick helped reshape genomic medicine at BWH. As founding director of Brigham Genomic Medicine (BGM), he built a pioneering program that united clinical care, research, and education to bring the power of genomics to patients with rare and undiagnosed conditions. He envisioned a future where precision medicine thrived not only on cutting-edge technology but also through a vibrant culture of collaboration, where clinicians, genetic counselors, laboratory scientists, and bioinformaticians worked together as equals. Under his leadership, BGM became a national model for translational genomics. Weekly multidisciplinary conferences and an innovative “crowdsourcing” strategy — engaging more than 50 experts across all the Harvard teaching hospitals — brought creative solutions to the most challenging cases. The impact was profound: In its first 100 referrals, BGM achieved a diagnostic yield of nearly 30% and uncovered dozens of previously unknown disease-associated genes. These breakthroughs not only provided long-awaited answers to patients and families but also advanced the broader understanding of human biology and rare diseases. Dick’s influence extended nationally through his leadership roles in the NIH Undiagnosed Diseases Network and in FaceBase, a consortium dedicated to elucidating the genetic basis of craniofacial birth defects. He helped establish rigorous protocols for deep phenotyping, advanced genomic analysis, and multidisciplinary case review. These frameworks have enabled diagnoses for patients who had exhausted all other avenues, underscoring his commitment to patient advocacy and scientific rigor.

Moreover, Dick was a passionate and impactful supporter of physician-scientist training and clinical genetics education. In 2000, he conceived of and planned the original MD-PhD summer course for the Harvard-MIT MD-PhD program, titled “Molecular Biology of Human Disease.” He directed this course for five years, which exposes all incoming MD-PhD students at the Harvard-MIT program to disease phenotypes and pathogenesis combined with introductory laboratory methods. The longevity and enduring success of this course, which is now titled “Investigations of Human Disease,” speaks to Dick’s vision of how to capture the imagination and dreams of students in their first month in medical school by connecting basic investigation of pathophysiology with clinical diseases and their phenotypes at the bedside. That same desire to create an arc between the bedside and the laboratory drove Dick to initiate a combined Internal Medicine and Medical Genetics residency program at BWH. Together with his colleague, Joel Katz, then the residency program director in internal medicine, they established the third program in the nation to obtain approval for a combined training program. The curriculum included unique clinical experiences in both ambulatory and inpatient settings, combined with instructive didactics at a time when the human genome had just been sequenced, and high-throughput sequencing of human genomes soon became possible. The graduates from this program are now impactful genetics researchers in many academic settings across the country, ensuring a lasting legacy of this training. In addition to these pivotal leadership roles in physician-scientist education, Dick actively supported and served as a resource for clinical and laboratory genetics fellows across the Harvard Medical School Genetics Training Program. All these programs have helped launch the careers of many leaders in the field of medical genetics.

Furthermore, Dick was an extraordinary leader and mentor whose influence shaped the culture and trajectory of the BWH Division of Genetics for more than two decades. As division chief, he recruited a slew of top-notch scientists across a wide range of biomedical science. Some of the current luminaries include Martha Bulyk, Karen Cichowski, and Vadim Gladyshev. Dick led the division with humility, vision, and an unwavering commitment to excellence. He believed that science thrives best in an environment of curiosity, generosity, and shared purpose, and he cultivated exactly that within the division. Colleagues and trainees often remarked that Dick created not just a workplace but a true intellectual home, one defined by collaboration, mutual respect, and kindness. He had an exceptional gift for recognizing potential in others and guiding them toward their highest aspirations. What set Dick apart as a leader was his belief that success was something to be shared. He took genuine pride in the achievements of his colleagues and trainees, celebrating their discoveries as if they were his own. He listened with patience, guided with wisdom, and stood beside others during moments of challenge or doubt. Through his quiet confidence and unfailing generosity, he inspired those around him to reach higher, think more deeply, and lead with integrity. Many who worked with him, including these authors, credit their professional growth and enduring sense of purpose to his mentorship, his belief in their potential, and his ability to create a community that balanced scientific rigor with humanity. His leadership left an indelible mark on the BWH Division of Genetics, one that continues to shape its values, its people, and its impact on the world of genomic medicine. Dick is survived by his beloved wife, Dr. Hao Wu, Professor at Harvard Medical School and Boston Children’s Hospital, and his children, Gordon and Alison.

## Figures and Tables

**Figure 1:**
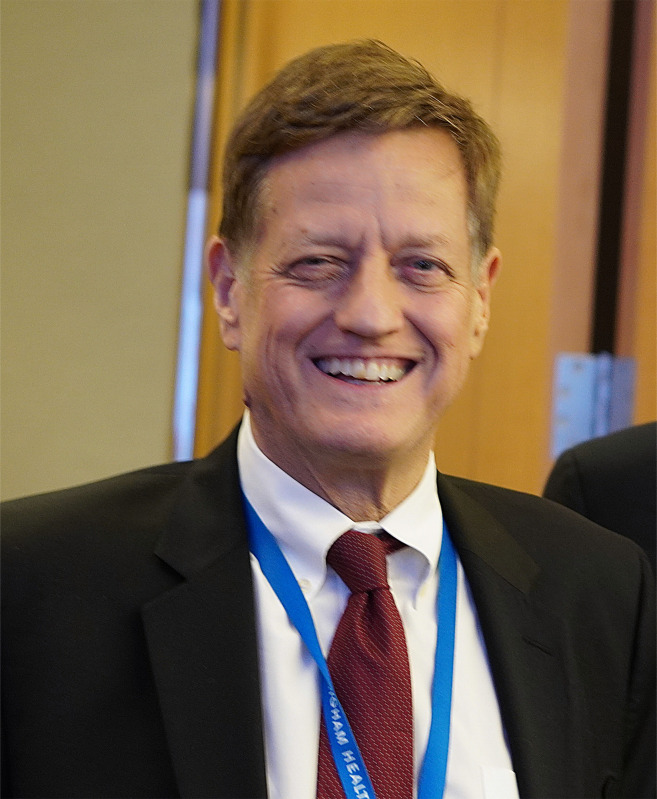
Richard L. Maas, MD, PhD. Image credit: Alireza Haghighi.

## Version 1 11/17/2025

Electronic publication
